# Nicotine Acts on Cholinergic Signaling Mechanisms to Directly Modulate Choroid Plexus Function

**DOI:** 10.1523/ENEURO.0051-19.2019

**Published:** 2019-04-23

**Authors:** Valeria Lallai, Nickolas Grimes, James P. Fowler, P. Adolfo Sequeira, Preston Cartagena, Agenor Limon, Margaret Coutts, Edwin S. Monuki, William Bunney, Angelo Demuro, Christie D. Fowler

**Affiliations:** 1 Department of Neurobiology and Behavior, University of California Irvine, Irvine, CA, 92697; 2 Department of Psychiatry and Human Behavior, School of Medicine, University of California Irvine, Irvine, CA 92697; 3 Department of Pathology, School of Medicine, University of California Irvine, Irvine, CA, 92697

**Keywords:** choroid plexus, drug dependence, microRNA, nicotine, nicotinic acetylcholine receptors, transthyretin

## Abstract

Neuronal cholinergic circuits have been implicated in cognitive function and neurological disease, but the role of cholinergic signaling in other cellular populations within the brain has not been as fully defined. Here, we show that cholinergic signaling mechanisms are involved in mediating the function of the choroid plexus, the brain structure responsible for generating CSF and releasing various factors into the brain. The choroid plexus was found to express markers of endogenous cholinergic signaling, including multiple nicotinic acetylcholine receptor (nAChR) subtypes in a region-specific manner, and application of nicotine was found to induce cellular activation, as evidenced by calcium influx in primary tissue. During intravenous nicotine self-administration in male rats, nicotine increased expression of transthyretin, a protein selectively produced and released by the choroid plexus, and microRNA-204 (mir-204), a transcript found in high levels in the choroid plexus and CSF. Finally, human choroid plexus tissue from both sexes was found to exhibit similar nAChR, transthyretin and mir-204 expression profiles, supporting the translational relevance of the findings. Together, these studies demonstrate functionally active cholinergic signaling mechanisms in the choroid plexus, the resulting effects on transthyretin and mir-204 expression, and reveal the direct mechanism by which nicotine modulates function of this tissue.

## Significance Statement

Tobacco/nicotine dependence is the largest preventable cause of disease and death worldwide. The current investigations establish the presence of cholinergic signaling mechanisms in the choroid plexus and demonstrate nicotine-mediated changes in transthyretin and microRNA-204 (mir-204) expression. These changes were attributed to nicotine's direct actions on nicotinic acetylcholine receptors (nAChRs) in the choroid plexus tissue. Therefore, these studies elucidate a previously unrecognized cholinergic neuroregulatory system, which may have relevant implications for disease states characterized by cholinergic dysfunction, such as Alzheimer’s disease, as well as nicotine dependence.

## Introduction

Cholinergic neuronal signaling has been shown to modulate various aspects of cognition, motivated behavior, and learning and memory function (Picciotto et al., 2012). At the cellular level, acetylcholine activates nicotinic acetylcholine receptors (nAChRs) or muscarinic AChRs. Such receptor activation has been shown to modulate a variety of cellular signaling processes, including neuronal excitability, gene expression, presynaptic release of neurotransmitters, and synaptic plasticity (Gray et al., 1996; Grady et al., 2001; Parikh et al., 2007; Dani et al., 2011). In addition to normal physiological function, cholinergic signaling has been associated with several neuropsychiatric and neurodegenerative disorders, such as dementia, Alzheimer’s disease, Parkinson’s disease, and schizophrenia, in which a deficit in cholinergic function is proposed to underlie disease-specific symptomology (Whitehouse et al., 1982; Coyle et al., 1983; Bordia et al., 2007; Bohnen and Albin, 2011; Sarter et al., 2012). Pharmacological approaches targeting the cholinergic system have also been the focus of multiple drug development efforts in the clinical setting (Jorenby et al., 2006; McHardy et al., 2017), and nicotine, the main psychoactive substance in tobacco and e-cigarettes, directly acts on nAChRs to mediate various aspects of drug dependence (Picciotto et al., 1998; Fowler et al., 2011; Fowler and Kenny, 2014).

The choroid plexus is a structure comprised of epithelial cells, which is localized throughout the ventricular system of the brain. Historically, the main role of the choroid plexus was attributed to the production of CSF. However, more recent findings have begun to elucidate other functions. For instance, CSF released from the choroid plexus has been shown to regulate neurogenesis throughout the lifespan (Lehtinen and Walsh, 2011; Lehtinen et al., 2011) and to play a role in Alzheimer’s disease pathology (Bateman et al., 2006; Krzyzanowska and Carro, 2012; Potter et al., 2013). The CSF-derived factors involved in these functions may include growth factors (e.g., Igf2, BDNF, and VEGF), proteins (e.g., transthyretin and apolipoprotein J), and could also include extracellular vesicles containing various RNA species (Lehtinen et al., 2011; Krzyzanowska and Carro, 2012; Ochoa et al., 2015; Lee et al., 2016; Riancho et al., 2017; Derkow et al., 2018), although the full extent of factors released by the choroid plexus with relevance to disease pathology remains to be elucidated. The extensive basolateral infoldings of the choroid plexus afford significant surface area for the release and uptake of signaling factors, whereas tight gap junctions and transporters regulate passage of substances between the blood and CSF (Balusu et al., 2016). The rate of production of CSF is projected to allow for turnover of approximately four times per day in humans (Johanson et al., 2005), and thus, the levels of circulating factors can be continuously regulated to influence neural function. Choroid plexus epithelium is found throughout the ventricular locations, including the lateral, third and fourth ventricles. At each of these sites during development, the choroid plexus has been proposed to differ in structure and function (Johanson et al., 2005; Lun et al., 2015a). While nicotine has been shown to be localized in choroid plexus tissue *in vitro* (Spector and Goldberg, 1982), it has remained unknown as to whether nAChRs are expressed and/or functionally mediate signaling within the choroid plexus cells.

In the current studies, we sought to identify whether endogenous cholinergic signaling mechanisms are localized in choroid plexus epithelium. Initial evidence indicated that nicotine could act directly to mediate choroid plexus function, and thus, we assessed whether cholinergic cells and nAChR subunits are differentially expressed across ventricular locations. Thereafter, we examined whether chronic nicotine self-administration would lead to altered expression of mRNA and miRNA in a region-specific manner. Specifically, we focused our investigations on the choroid plexus-specific protein, transthyretin, which has been found to be increased in the CSF during nicotine exposure, and microRNA-204 (mir-204), which is expressed in high density in the choroid plexus (Li et al., 2000, 2016; Oberwinkler et al., 2005). Primary choroid plexus cell culture was used to provide further evidence of nicotine’s direct impact on cellular activation with calcium imaging. Finally, nAChR subunits, transthyretin and mir-204 expression were examined in tissue from human subjects, thus providing translational relevance for the potential impact of these findings in humans.

## Materials and Methods

### Animals

Adult, male Wistar rats (RRID:RGD_13508588; *n* = 78) weighing 275–300 g were obtained from Charles River Laboratories and housed two to three per cage. ChAT-IRES-Cre (B6;129S6-Chattm2(cre)Lowl/J; Stock 006410; RRID:IMSR_JAX:006410) and ROSA^26Sor^-tdTomato reporter (B6.Cg-Gt(ROSA)26Sortm14(CAG-tdTomato)Hze/J; Stock 007914; RRID:IMSR_JAX:007914) mice were obtained from The Jackson Laboratory and bred within our colony as hemizygous mating pairs. Both founder lines were backcrossed on a C57BL/6J background (RRID:IMSR_JAX:000664) for at least 10 generations before the current breeding cross. Pups were weaned at 21 d of age and housed three to five per cage. Adult male hemizygous *ChAT-IRES-Cre::Rosa-TdTomato* mice (*n* = 5) were used to characterize the presence of cholinergic cells within the choroid plexus. Rats and mice were maintained in an environmentally controlled vivarium on a 12/12 h light/dark cycle, and food and water were provided ad libitum. All experimental subjects were randomly assigned to treatment conditions. However, during self-administration, rats were mildly food restricted to 85–90% of their free-feeding body weight, while water was maintained *ad libitum*. All procedures were conducted in strict accordance with ethical regulations outlined in the National Institutes of Health Guide for the Care and Use of Laboratory Animals and were approved by the Institutional Animal Care and Use Committee at the University of California, Irvine.

### Mouse genotyping

At 21 d of age, mouse pups were weaned and their tails were clipped for genetic analysis. Subjects were genotyped by PCR with the following primers: *ChAT-IRES-Cre*: 5’-GTT TGC AGA AGC GGT GGG-3’ (wild type forward), 5’-CCT TCT ATC GCC TTC TTG ACG-3’ (mutant forward), 5’-AGA TAG ATA ATG AGA GGC TC-3’ (common reverse); *Rosa-TdTomato* 5’-AAG GGA GCT GCA GTG GAG TA-3’ (wild type forward), 5’-CCG AAA ATC TGT GGG AAG TC-3’ (wild type reverse), 5’-CTG TTC CTG TAC GGC ATG G-3’ (mutant forward), 5’-GGC ATT AAA GCA GCG TAT CC-3’ (mutant reverse).

### Drugs

Mecamylamine hydrochloride (Tocris Bioscence) was diluted in 0.9% sterile saline and injected subcutaneously at a dose of 2 mg/kg. (-)-Nicotine hydrogen tartrate salt (MP Biomedicals) was dissolved in 0.9% sterile saline (pH 7.4), and doses of nicotine refer to the free-base form. Ketamine (KetaVed, Patterson Veterinary) and xylazine (AnaSed, Patterson Veterinary) were diluted in sterile saline and simultaneously injected intraperitoneally at a dose of 100 and 10 mg/kg, respectively, for mouse perfusions. Isoflurane (IsoFlo, Abbott Laboratories) was administered as a 1–3% mixture with oxygen via inhalation.

### Experimental design

#### Mouse perfusion and tissue processing

Adult, hemizygous ChAT-IRES-Cre::Rosa-TdTomato mice were anesthetized with ketamine/xylazine and perfused through the ascending aorta with 0.9% saline, followed by 4% paraformaldehyde in 0.1 M PBS (pH 7.4). Brains were harvested, postfixed for 2 h in 4% paraformaldehyde, and then stored in 30% sucrose in PBS. After at least 72 h, brains were cut into 30-μm coronal sections on a cryostat, and floating sections were stored in 0.1 M PBS with 0.01% sodium azide at 4**°**C. To visualize TdTomato fluorescence, sections were directly mounted onto slides and coverslipped with vectashield.

#### Primary choroid plexus epithelial cell culture and Ca^2+^ fluorescence imaging

Primary cultures of choroid plexus epithelial cells were prepared from dissected tissue of naïve, adult Wistar male rats (*n* = 6). Directly after decapitation, choroid plexus tissue was separately isolated from the third ventricle (3V) of the brain and then pooled across subjects to derive sufficient cell quantity for primary culture. Thereafter, cells were treated with collagenase Type II and TrypLE Express (Gibco). To obtain a monolayer culture, cells were plated on a modified recording chamber consisting of a silicon O-ring (Sealing Devices Incorporation), mounted on a cover glass (Warner Instruments), and treated with poly-D-lysine coated to improve cells adhesion. Cultures achieved confluency by 2–3 d at ≅10,000 cells/cm^2^ in DMEM with 10% FBS exosome-depleted serum and 1× pen-strep solution (Gibco). Culture media was changed every 2–3 d, unless otherwise specified. Cell culture plates were maintained in a humidified 37°C incubator containing 5% CO_2_. The Ca^2+^-sensitive dye Cal-520 (AAT Bioquest) was reconstituted with DMSO containing 20% pluronic F-127 (Invitrogen). Before imaging, culture medium was replaced with a Ca^2+^-containing HEPES-buffered salt solution composed of 135 mM NaCl, 5.4 mM KCl, 2 mM CaCl_2_, 1 mM MgCl_2_, 10 mM HEPES, and 10 mM glucose (pH 7.4). Cells were then incubated with 5 μM Cal-520 (AM esters) for ∼50 min at room temperature. Cells were washed afterward for at least 30 min, and then nicotine was applied by pipetting a fixed aliquot (30 μl) of the diluted stock solution into the recording chamber (470-μl volume) directly above the microscope objective. The EC50 for nicotine/ACh for the different neuronal nAChRs subtypes range from ∼2 to 30 μM (Demuro et al., 2001; Demuro and Parker, 2005). As our rationale was to generate a robust response by activating all the possible nAChRs subtypes present in the cells, we performed experiments using 30 μM nicotine. The imaging system consisted of an inverted microscope (Olympus IX 71) equipped with a 60× total internal reflection fluorescence (TIRF) oil immersion objective (NA 1.45) as previously described (Demuro and Parker, 2005). Fluorescence excitation was induced with a 488-nm laser from a laser combiner (L4C OXXIUS S.A.). Emitted fluorescence (510 nm) was detected with a green bandpass (520 ± 20 nm) emission filter to record Ca^2+^ fluorescence signals generated by Cal-520. Images were captured using a sCMOS camera (Andor Zyla 4.2) providing a final magnification of 200 nm per pixel. Image acquisition was controlled by MicroManager open source microscopy software (https://micro-manager.org/) connected to the computer trough a USB2 port. The camera was set to acquire from a central region of interest of 256 × 256 pixels, corresponding to ∼50 × 50 μm of the specimen. Time-lapse images (frame rate 50 fps) were captured using Micromanager software. Images were processed using MetaMorph software package (Universal Imaging) and measurements were exported to Microcal Origin2017 (OriginLab) for analysis and graphing. Averaged fluorescence intensities were measured from regions of interest and expressed as a pseudo ratio (Δ*F*/*Fo*) of the change in fluorescence (Δ*F*) divided by the resting fluorescence before treatment (*Fo*).

#### Intravenous nicotine and saline self-administration

Before catheter implantation, rats were food restricted to 85–90% of their free-feeding body weight and trained to press an active lever in an operant chamber (Med Associates) for food pellets (45 mg, 5TUM, TestDiet). Rats were trained up to a fixed-ratio 5 time out 20 s (FR5TO20) schedule of reinforcement during 1-h sessions. Self-administration sessions were performed using two retractable levers (one active, one inactive) that extend 1cm into the chamber. Completion of the response criteria on the active lever resulted in delivery of the food pellet. Responses to the inactive lever resulted in no scheduled consequence. After achieving food-training criteria of >60 pellets per session for three consecutive days, rats were implanted with the intravenous catheters. For intravenous surgery, rats were placed under anesthesia with an isoflurane (1–3%)/oxygen mixture and surgerized as described previously (Fowler et al., 2011). Catheters were flushed daily via the port on the rat’s dorsal region with heparin diluted in physiologic sterile saline solution. After >48 h of postsurgical recovery, rats were food restricted as above and permitted to respond for food reinforcement under the FR5TO20 schedule. Subjects were randomly assigned into either the nicotine or saline condition (total *n* = 44 nicotine; *n* = 28 saline). After reinstating food responding across three sessions, rats in the nicotine group were then permitted access to the 0.03 mg kg^−1^ training dose of nicotine for seven sessions and then transitioned to a higher dose of nicotine (0.12 mg kg^−1^), whereas the saline control were maintained on saline. To probe for the involvement of nAChR signaling in mediating changes in transthyretin expression, 20 min before the last session, rats were injected with mecamylamine (2 mg/kg, s.c.) or saline (s.c.). The specific sequence of testing was as follows: food training (5+ sessions until criterion is achieved) → intravenous surgery and recovery → reinstated food training (two to three sessions) → 0.03 mg/kg/infusion nicotine (7 sessions) → 0.12 mg kg^−1^ per infusion nicotine (1 session; for the mecamylamine study, vehicle/mecamylamine administered before this session). We designed the experiment with the transition to this moderately-high dose to have the rats actively titrate their behavior to obtain the desired amount of nicotine. In a prior study (Fowler et al., 2011), we found that rats will titrate their nicotine intake to a similar level across the dose range of 0.06–0.18 mg kg^−1^ per infusion, and given the localization of the dorsal 3V (d3V) choroid plexus in juxtaposition to the medial habenula, which is preferentially activated at higher nicotine doses (Fowler et al., 2011; Frahm et al., 2011), we hypothesized that this dose may be particularly relevant to reveal functionally significant processes potentially mitigating drug intake. Subjects in the saline self-administration control group went through the identical procedure as noted above, but saline was provided for intravenous self-administration in the absence of nicotine. Subjects were killed after the final self-administration session, and saline and mecamylamine-injected subjects were pseudo-yoked to the number of infusions of the nicotine subjects to normalize for the amount of total saline intake between groups. All subjects were within a proximate range for the amount of saline infused via the intravenous catheter.

#### Choroid plexus tissue dissection

Rats were placed under isoflurane anesthesia and decapitated. The whole brain was removed and immediately transferred to a Petri dish and cut along the coronal plane at the levels of the septum and raphe nucleus with a straight edge blade. The dorsal brain portion was transferred to a Petri dish and superglued to the bottom of the dish, with the cerebellum and brainstem facing upward. The dish was filled with ice cold 0.1 M PBS and placed under a dissecting microscope. Thereafter, the cerebellum was gently separated from the cerebrum tissue with forceps, allowing the fourth ventricle to be exposed. The choroid plexus was gently removed and verified to not contain any attached brain tissue or dura matter via microscopic inspection. For the lateral and third choroid plexus dissections, the middle portion of the brain was superglued on the ventral surface to a Petri dish, and the dish was filled with ice cold PBS and placed under the dissecting microscope. A straight edge blade was used to cut the corpus callosum and hippocampus along the longitudinal fissure. Thereafter, the cortex and hippocampus were gently pulled to either lateral side with forceps to expose the d3V choroid plexus, which was removed and visually inspected under the microscope to ensure only the presence of the choroid plexus tissue (no attached brain tissue). Next, to obtain the lateral ventricle choroid plexus, the hippocampus was dislodged on each side to reveal the lateral ventricles, and the choroid plexus was then removed from this location. Further, the hippocampus and habenula were visually inspected under the microscope to ensure that they were fully intact, and these tissues were then biobanked. Samples were flash frozen in a collection tube and coded with unique numbers so that experimenters processing the tissue were blinded to the experimental condition. Samples were assigned in a random within group manner into analyses for RT-PCR by a second experimenter while maintaining blinding for the experimenter processing the tissue, and each sample was analyzed for multiple PCR assays based on the amount of RNA derived from each subject. Postmortem human choroid plexus samples were immediately frozen on dry ice at the time of dissection and placed in coded collection containers. All tissue was stored at –80°C.

#### Human brain tissue

Postmortem human choroid plexus tissue was provided by the University of California Irvin Brain Bank through a collaboration. Subjects ranged in age from 49 to 59, consisted of two men and one woman, and were classified as “control” subjects (although history of multi-drug use was noted in one of the men and the woman; the woman was also diagnosed with obesity). Experimenters did not receive access to personal identifying information regarding the human subjects. Brain tissue collection was conducted in accordance with the University of California Irvin Brain Bank standard operating procedure, relevant ethical regulations, and approved human Institutional Review Board protocol at University of California Irvine. Informed consent was provided by the legal next-of-kin with a University of California Irvine institutional approved consent form.

### RNA analysis

RNA was extracted from homogenized tissue with TRIzol reagent (Ambion Life Technologies) via the manufacturer’s protocol. The quality of the RNA was determined by a NanoDrop 2000 spectrophotometer (ThermoScientific). For each sample, 100 ng of total RNA was reverse transcribed into cDNA with the iScript cDNA synthesis kit (Bio-Rad Laboratories). RT-qPCR was performed for nAChR subunits, transthyretin (TTR), mir-204 and the housekeeping genes, *β-actin* (ACTB) or *U6* (RNU6). TaqMan Universal Master Mix II with real-time PCR gene expression assays for CHRNA3, CHRNA5, CHRNA7, CHRNA4, CHRNA6, CHRNA9, CHRNA10, CHRNB4, CHRNB2, CHRNB3, TTR, mir-204, and ACTB (control) or microRNA assay for miR-204 and RNU6 (control) were used according to manufactures parameters (Applied Biosystems). Samples were tested in duplicate or triplicate (depending on quantity of RNA available per dissection) and quantified with a CFX96 RT-qPCR system (Bio-Rad). Samples with Ct values >35 cycles were considered outside of the range of inclusion as predetermined criteria, and thus, these samples were determined to exhibit no RNA expression in the tissue of interest. Normalized gene expression (2^ΔCt^) was calculated with the equation 2^^(β-actin Ct – target mRNA Ct)^. For nAChR subunit genes, normalized values were multiplied by 10,000 to represent data as whole numbers, and for the miRNA gene expression assay, normalized values were multiplied by 1000. For the microRNA assay, normalized mi-204 expression (2^ΔCt^) was calculated with the equation 2^^(U6 Ct – target miRNA Ct)^. After samples were processed, group assignment was revealed to permit comparisons of the data.

### Statistical analysis

Statistical details of experiments can be found in the results and figure legends. Data were analyzed by *t* test (two-sided, unpaired) or one-way ANOVA, followed by a Bonferroni *post hoc* test with correction for multiple comparisons (GraphPad Prism 6), as appropriate. The criterion for significance was set at *p* < 0.05.

## Results

### Cholinergic signaling mechanisms are localized in epithelial cells of the choroid plexus

**Figure 1.**
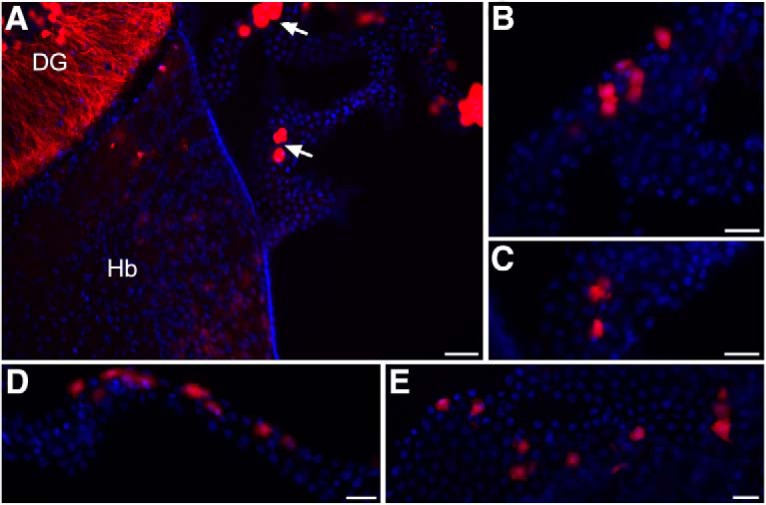
Cholinergic choroid plexus epithelial cells. ***A***, Visualization of cholinergic cells in transgenic mice expressing td-Tomato fluorescence under the ChAT promotor. Cholinergic cells (red) are found within the dentate gyrus of the hippocampus (DG), habenula (Hb), and the choroid plexus of the d3V (arrows). ChAT: red; DAPI nuclei: blue. Scale bar = 50 μm. ***B***, Higher magnification of the choroid plexus epithelium from the top arrow in panel ***A*** illustrates scattered expression pattern with groups of adjacent cholinergic-positive cells. Scale bar = 25 μm. ***C***, Higher magnification image of the epithelium from the bottom arrow in panel ***A***. Scale bar = 25 μm. ***D***, Cholinergic-positive cells were also identified in the lateral ventricle with a similar expression pattern. Scale bar = 25 μm. ***E***, In the fourth ventricle choroid plexus, similar ChAT expression was observed in a subset of epithelial cells. Scale bar = 25 μm.

In *ChAT-IRES-Cre::ROSA*^*26Sor*^*-tdTomato* mice, we identified choroid plexus epithelial cells that express *ChAT*, the conventional marker used to identify cholinergic cells in the brain. Other brain region specific cholinergic expression in these mice was representative of that described in the literature and as previously characterized in this mouse line (GENSAT, 2019; Chen et al., 2018), for instance as shown in hippocampus and habenula (Fig. 1*A*). Within the d3V, scattered cholinergic cells were found throughout the choroid plexus (Fig. 1*A–C*). Further, choroid plexus epithelial cells in the lateral ventricle (Fig. 1*D*) and fourth ventricle (Fig. 1*E*) also exhibited a similar expression profile with a subset of cholinergic-positive cells.

### Nicotine directly activates a subset of epithelial cells, inducing calcium influx

**Figure 2.**
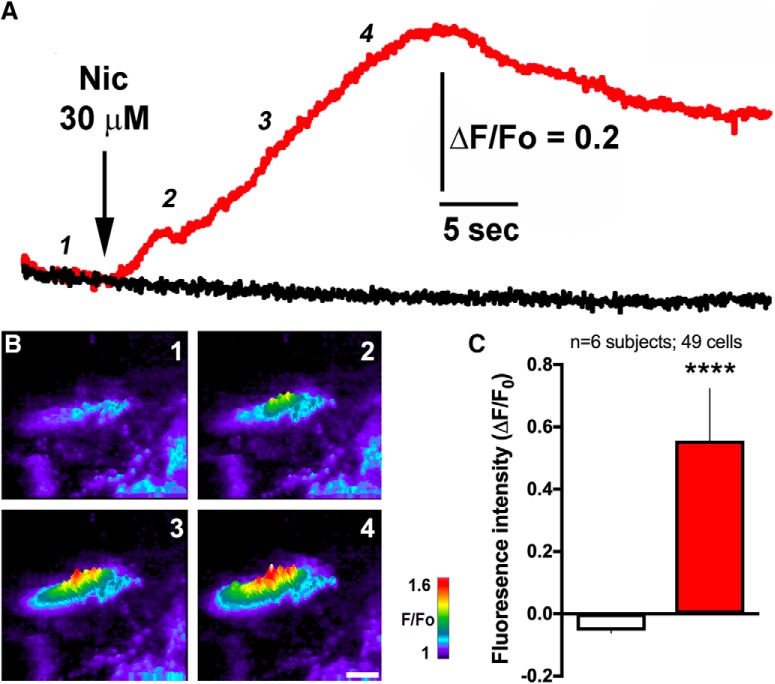
Nicotine activates calcium signaling in choroid plexus epithelial cells *in vitro*. ***A***, Time course of Ca^2+^-dependent fluorescence signal recorded from responding (red) and nonresponding (black) cells following application of 30 μM nicotine. Traces represent average intensity from nine responsive (red) and 40 nonresponsive (black) primary choroid plexus epithelial cells *in vitro*. Primary choroid plexus obtained from *n* = 6 rats (five cell culture plates analyzed, resulting in 49 cells quantified). ***B***, Representative images of a responsive cell (center) and nonresponsive cells (bottom right) captured at the time points 1–4 as indicated in ***A***. Increasing cytosolic free Ca^2+^ is represented by warmer colors (as depicted with the color bar) and increasing height of each pixel. Scale bar = 20 μm. ***C***, Average fluorescence signals measured at the peak of each recording obtained during 11 separated trials; ****p < 0.0001. Data represent mean ± SEM. Central tendency (mean) and variation (SEM) values for each are as follows: nonresponsive, –0.054 ± 0.008 and responsive, 0.556 ± 0.167.

To determine whether nicotine functionally activates the choroid plexus cells, as opposed to just being transported through the tissue (Spector and Goldberg, 1982), we performed TIRF Ca^2+^ imaging experiments to monitor Ca^2+^ influx into the cytosol during nicotine-mediated activation of nAChR channels in the plasma membrane. Application of nicotine (30 μM) into the bath solution triggered a time-dependent rise in Ca^2+^ fluorescence signal within a few seconds after administration (Fig. 2*A*,*B*). The emitted fluorescence signal slowly decayed during the 30-s recording window, which is in agreement with the nAChR kinetics for receptor activation followed by desensitization. Of note, a statistically significant robust response to nicotine was only found in a subset (18.4%) of the cells imaged (two-sided, unpaired *t* test, *t*_(47)_ = 7.79, *p* < 0.0001, *R* ^2^ = 56; Fig. 2*C*). The “nonresponder” cells did exhibit localized, low-intensity spontaneous intracellular Ca^2+^ signaling in each imaged field during the recording period, verifying that all the cells were sufficiently loaded with the calcium indicator Cal-520 (Fig. 2). Finally, cultured cells were tested with 100 mM ionomycin to further validate loading of the Cal-520 dye, and all visualized cells responded with a high fluorescence signal (data not shown), verifying the dye presence and cellular functionality potential. Of further note, the partial permeability to Ca^2+^ for nAChRs subtypes ranges from 0.8 to 4 pS. This relatively small conductance allows a large flux of Ca^2+^ into the cells which, by binding to the Ca^2+^-sensitive dye, is amplified to become a robust fluorescence signal. As such, in the TIRF technique, it is now possible to monitor Ca^2+^ flux trough the opening of a single nAChRs (Demuro and Parker, 2005, 2006). Although we cannot exclude the possibility of other components contributing to the cytosolic Ca^2+^ rise, such as CICR through RyRs or calcium influx through store-operated Ca^2+^ channels, the temporal evolution and the time to response obtained in these experiments strongly suggest that the fluorescence signal is due to the opening nAChRs in the plasma membrane of these cells.

### Multiple nAChR subunits exhibit region-specific expression patterns in the choroid plexus

**Figure 3.**
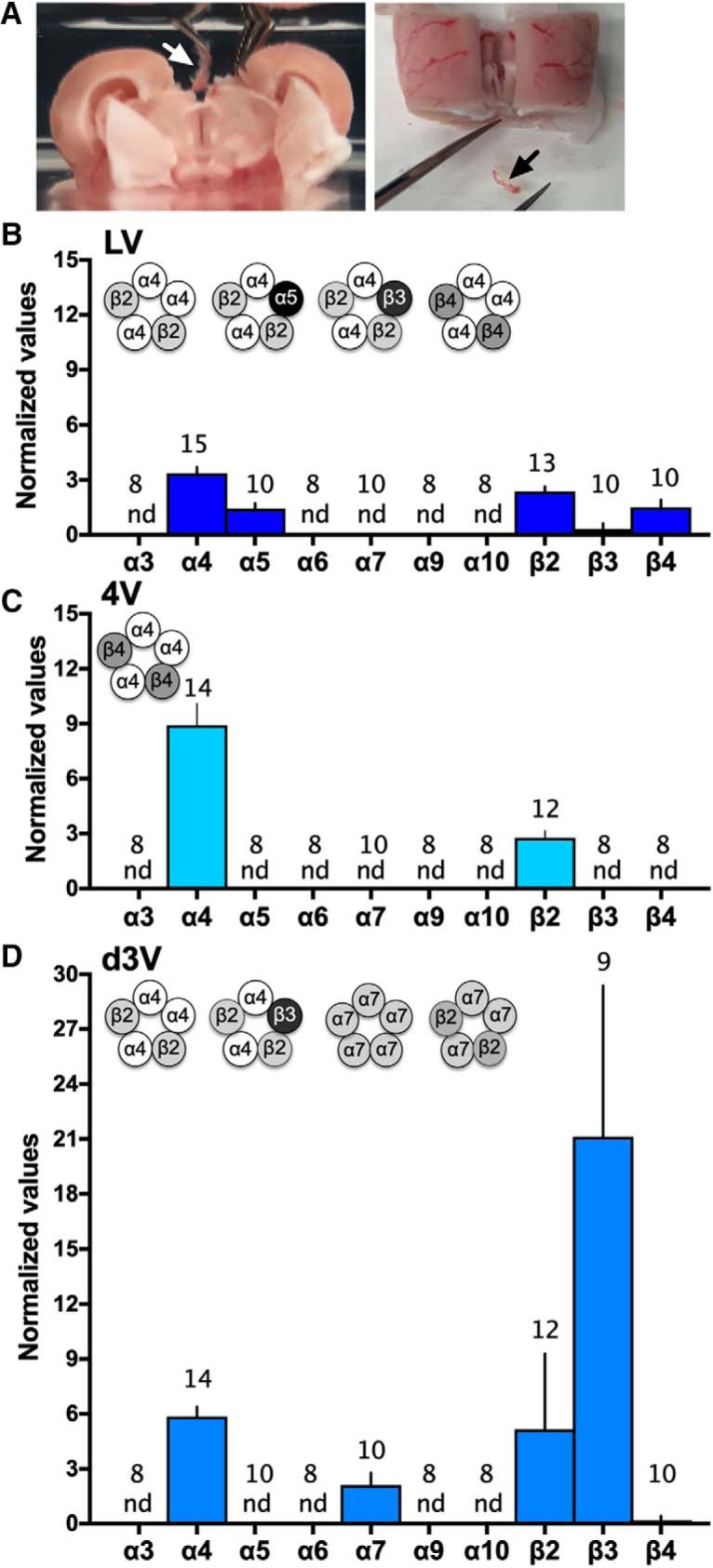
nAChR subunit expression in choroid plexus derived from the lateral, fourth, and d3Vs. Choroid plexus was discretely dissected from the ventricles of the brain. ***A***, The outer layer of cortex and hippocampus were gently separated from the midline to allow for visualization and removal of the d3V choroid plexus with visualization via dissection microscope (left image with white arrow), and following removal, the choroid plexus was clearly visualized as completely separate from brain tissue (right image with black arrow). Similar dissections were conducted with microscopic visualization and verification for the lateral and fourth ventricle locations. ***B–D***, The expression of nAChR subunits was examined in choroid plexus tissue from rats (*n* = 8–15/group, specific number per group denoted on each bar). ***B***, In the lateral ventricle (LV), mRNA expression was found for the α4 (*Chrna4*), α5 (*Chrna5*), β2 (*Chrnb2*), β3 (*Chrnb3*), and β4 (*Chrnb4*) nAChR subunits. Putative nAChR subtype schematics are graphically illustrated (insert). Statistical data are as follows [central tendency (mean) ± variation (SEM), lower 95% confidence interval, upper 95% confidence interval]: α4: 3.35 ± 0.34, 2.61, 4.09; α5: 1.44 ± 0.29, 0.77, 2.10; β2: 2.37 ± 0.28, 1.77, 2.97; β3: 0.32 ± 0.32, 0, 1.03; and β4: 1.50 ± 0.42, 0.56, 2.45. ***C***, In the fourth ventricle (4V), only α4 (*Chrna4*) and β2 (*Chrnb2*) mRNAs were detected, and thus, the α4β2 nAChR subtype may be present (schematic insert). Statistical data are (mean ± SEM, lower CI, upper CI): α4: 8.93 ± 1.18, 6.38, 11.47 and β2: 2.77 ± 0.38, 1.93, 3.61. ***D***, In the d3V, subunit expression consisted of α4 (*Chrna4*), α7 (*Chrna7*), β2 (*Chrnb2*), and β3 (*Chrnb3*), which allows for four different putative nAChR subtypes (schematic insert). Statistical data are (mean ± SEM, lower CI, upper CI): α4: 5.86 ± 0.53, 4.71, 7.01; α7: 2.12 ± 0.67, 0.61, 3.62; β2: 5.16 ± 1.19, 2.53, 7.79; and β: 21.12 ± 8.25, 2.09, 40.14. For each nAChR gene above, expression data were normalized to expression of β-actin as the endogenous control. nd = not detected based on the predetermined RT-qPCR criteria (Ct value > 35). Data represent mean normalized values ± SEM.

A variety of nAChR subunits have been documented within the brain, and functional pentameric nAChR subtypes include varying combinations of α2-α10 and/or β2-β4 subunits (Fowler et al., 2008). In the current studies, we systematically examined the expression of nAChR subunit mRNA in the three choroid plexus locations in rats. Tissues were discretely dissected from each ventricle location (e.g., as shown for the d3V; Fig. 3*A*). Interestingly, differential expression profiles were discovered based on the ventricular localization. In the lateral ventricle choroid plexus (Fig. 3*B*), α4, α5, β2, β3, and β4 nAChR subunits were found. In the fourth ventricle choroid plexus (Fig. 3*C*), α4 and β2 nAChR subunits were expressed. Finally, α4, α7, β2, and β3 nAChR subunits were identified in the d3V choroid plexus (Fig. 3*D*). Based on the literature for nAChR subunit stoichiometry, multiple functional receptor subtypes would be predicted across ventricular locations, as illustrated in the figure insets (Fig. 3*B–D*).

### The α4 and β2 nAChR subtypes are differentially expressed across ventricular sites and nicotine self-administration does not consistently alter subunit expression

**Figure 4.**
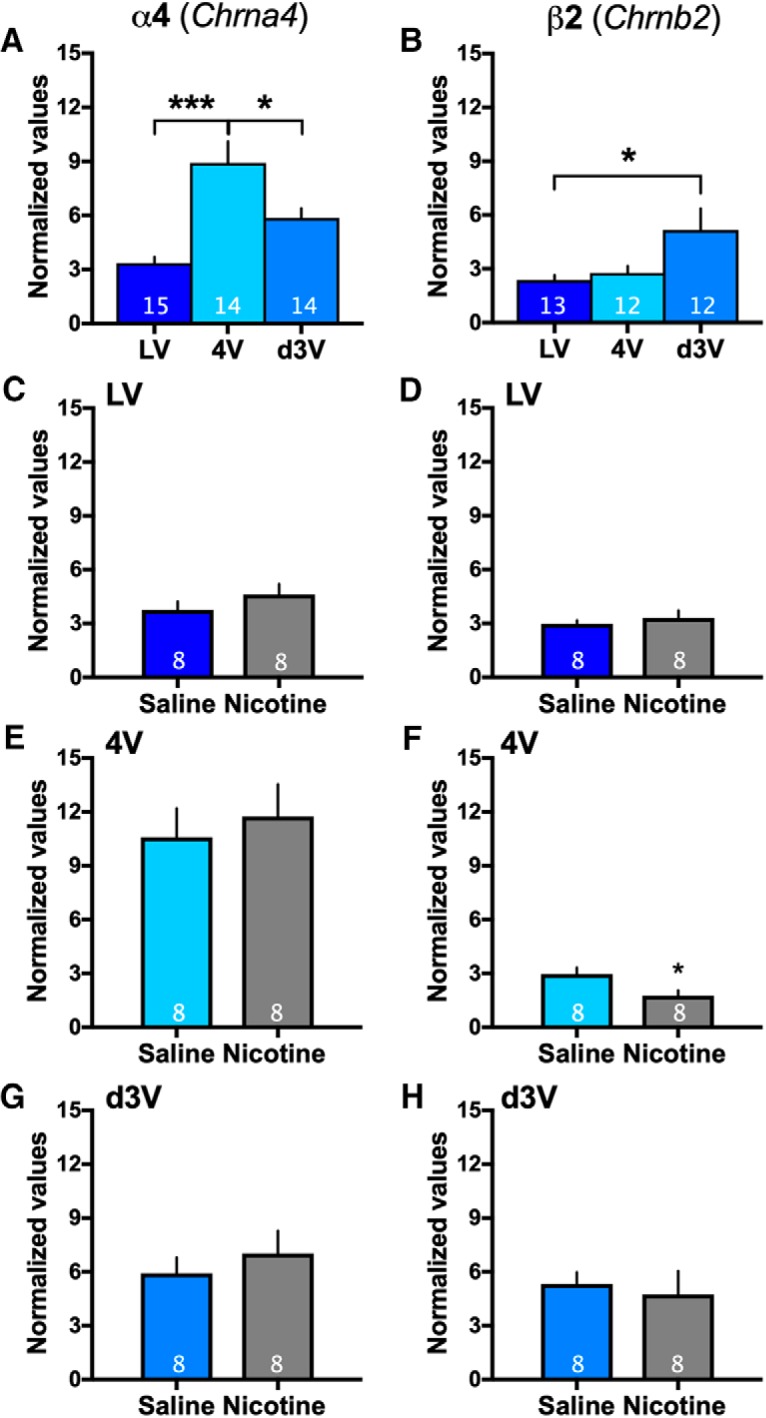
Differential expression of α4 and β2 nAChR subunits across choroid plexus sites. ***A***, ***B***, Given that α4 and β2 subunits were found in all choroid plexus sites, their relative expression was compared under baseline conditions (*n* = 12–15/group, specific number per group denoted on each bar). ***A***, For the α4 nAChR subunit, significantly increased expression was found in choroid plexus from the fourth ventricle compared to tissue from the lateral and d3V. *Post hoc* corrected for multiple comparisons; *p < 0.05, ***p < 0.001. Central tendency (mean) and variation (SEM) values for each are as follows: *LV*: 3.350 ± 0.34; *4V*: 8.925 ± 1.18; and *d3V*: 5.861 ± 0.53. For each nAChR gene, expression data were normalized to expression of β-actin. ***B***, With regard to the β2 nAChR subunit, significantly increased expression was found in the d3V choroid plexus as compared to the lateral ventricle (LV). *Post hoc* corrected for multiple comparisons; *p < 0.05. Central tendency (mean) and variation (SEM) values for each are as follows: *LV*: 2.371 ± 0.27; *4V*: 2.773 ± 0.38; and *d3V*: 5.156 ± 1.19). 4V: fourth ventricle. For each nAChR gene, expression data were normalized to expression of β-actin. Data represent mean normalized values ± SEM. ***C–H***, Expression of α4 and β2 nAChR subunits with nicotine or saline self-administration (*n* = 8/group as denoted on each bar). Following saline or nicotine self-administration in rats, normalized expression levels of α4 and β2 nAChR subunits were compared. For the α4 nAChR subunit (left panels), similar mRNA expression levels were found with saline and nicotine self-administration in choroid plexus from the (***C***) LV, (***E***) 4V, and (***G***) d3V. For the β2 nAChR subunit (right panels), differences were not found in mRNA expression in the (***D***) LV or (***H***) d3V, whereas a significant decrease with nicotine was found in the (***F***) 4V choroid plexus; *p < 0.05. Central tendency (mean) and variation (SEM) values (***C–H***) are as follows: *α4 LV - Saline*: 3.758 ± 0.46; *Nicotine*: 4.600 ± 0.60; *α4 4V - Saline*: 10.60 ± 1.60; *Nicotine*: 11.74 ± 1.79; *α4 d3V - Saline*: 5.915 ± 0.87; *Nicotine*: 7.015 ± 1.28; *β2 LV - Saline*: 2.978 ± 0.19; *Nicotine*: 3.310 ± 0.41; *β2 4V - Saline*: 2.953 ± 0.37; *Nicotine*: 1.762 ± 0.30; *β2 d3V - Saline*: 5.319 ± 0.66; and *Nicotine*: 4.732 ± 1.31. For each nAChR gene, expression data were normalized to expression of β-actin. Data represent mean normalized values ± SEM.

Given that α4 and β2 were common to all locations, we next examined whether the level of expression significantly varied between the sites. For the α4 nAChR subunit, statistically significant differences in expression were found between the ventricular locations (one-way ANOVA, *F*_(2,40)_ = 13.69, *p* < 0.0001, *R* ^2^ = 0.41; Fig. 4*A*). *Post hoc* analyses revealed increased α4 nAChR subunit mRNA expression in the fourth ventricle compared to the lateral and 3V. For the β2 nAChR subunit, significant differences were again found among the groups (one-way ANOVA, *F*_(2,34)_ = 4.30, *p* = 0.0217, *R* ^2^ = 0.20; Fig. 4*B*), with *post hoc* analyses revealing significantly higher β2 subunit mRNA expression in the 3V choroid plexus as compared to the lateral.

**Figure 5.**
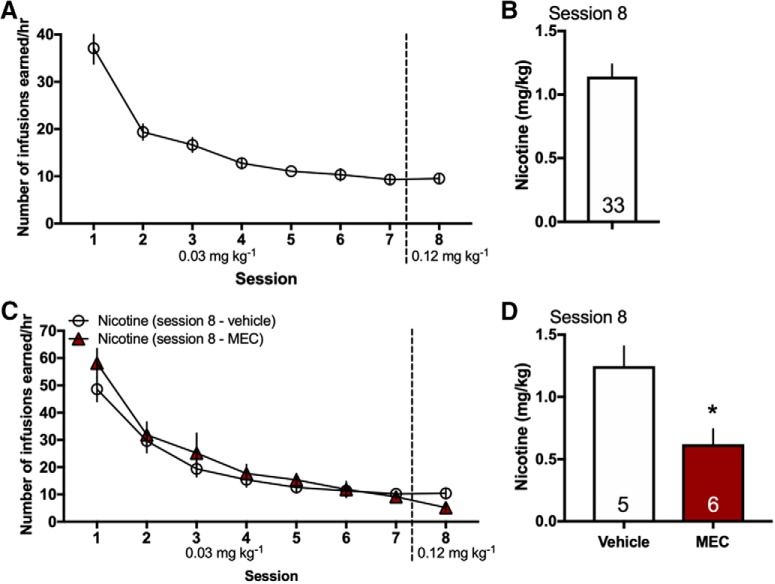
Intravenous nicotine self-administration in rats. Rats were trained in the intravenous nicotine self-administration protocol; access to the acquisition dose of 0.03 mg kg^−1^ per infusion was provided for 7 d, followed by 0.12 mg kg^−1^ per infusion on the eighth session. ***A***, The number of nicotine infusions across sessions during nicotine self-administration corresponds to tissue analyzed for studies shown in Figures 4, 6, 7 (*n* = 33). ***B***, On the final session, the subjects shown in Figure 5*A* self-administered a mean of 1.1 mg/kg nicotine (±0.09 SEM). ***C***, For the data presented in Figure 6*C*, groups were tested across doses as described above but were administered either vehicle or mecamylamine before the self-administration session on session 8 (*n* = 5–6/group). ***D***, For session 8, a significant decrease in the amount of nicotine consumed was found in the mecamylamine group compared to the vehicle group; *p < 0.05. The mean nicotine intake (mg/kg) ± SEM for each group was: *Vehicle*: 1.248 ± 0.16 and *Mecamylamine*: 0.62 ± 0.122. Specific subject numbers per group are denoted on the bar graphs. Data represent mean values ± SEM.

In prior studies, chronic nicotine exposure has been found to increase radiotracer agonist binding for nAChRs in human smokers and rodent models (Benwell et al., 1988; Marks et al., 1992; Breese et al., 1997), suggesting a functional upregulation of receptors. Thus, given the differences found in the expression pattern of α4 and β2 nAChR subunits across ventricular choroid plexus sites, we next sought to examine whether chronic nicotine intake would alter the mRNA expression of these subunits. Rats were behaviorally tested in the intravenous nicotine self-administration protocol to obtain tissue for analysis (Fig. 5*A*,*B*), and saline self-administration subjects were pseudo-yoked to allow for similar levels of saline intake between groups. When comparing tissue from saline versus nicotine self-administering rats, statistically significant differences were not found for the α4 nAChR subunit in the choroid plexus across locations, including the lateral ventricle (two-sided, unpaired *t* test, *t*_(14)_ = 1.11, *p* = 0.29, *R* ^2^ = 0.08; Fig. 4*C*), fourth ventricle (two-sided, unpaired *t* test, *t*_(14)_ = 0.47, *p* = 0.64, *R* ^2^ = 0.02; Fig. 4*E*), and d3V (two-sided, unpaired *t* test, *t*_(14)_ = 0.71, *p* = 0.49, *R* ^2^ = 0.03; Fig. 4*G*). For the β2 nAChR subunit, significant differences between groups were also not found in the lateral ventricle site (two-sided, unpaired *t* test, *t*_(14)_ = 0.47, *p* = 0.47, *R* ^2^ = 0.04; Fig. 4*D*) and d3V sites (two-sided, unpaired *t* test, *t*_(14)_ = 0.40, *p* = 0.70, *R* ^2^ = 0.01; Fig. 4*H*). However, the fourth ventricle choroid plexus comparison did reveal a decrease in β2 nAChR mRNA with nicotine self-administration (two-sided, unpaired *t* test, *t*_(14)_ = 2.52, *p* = 0.0245, *R* ^2^ = 0.31; Fig. 4*F*).

### Nicotine self-administration selectively increases the mRNA expression of the choroid plexus-specific protein, transthyretin, in the d3V

**Figure 6.**
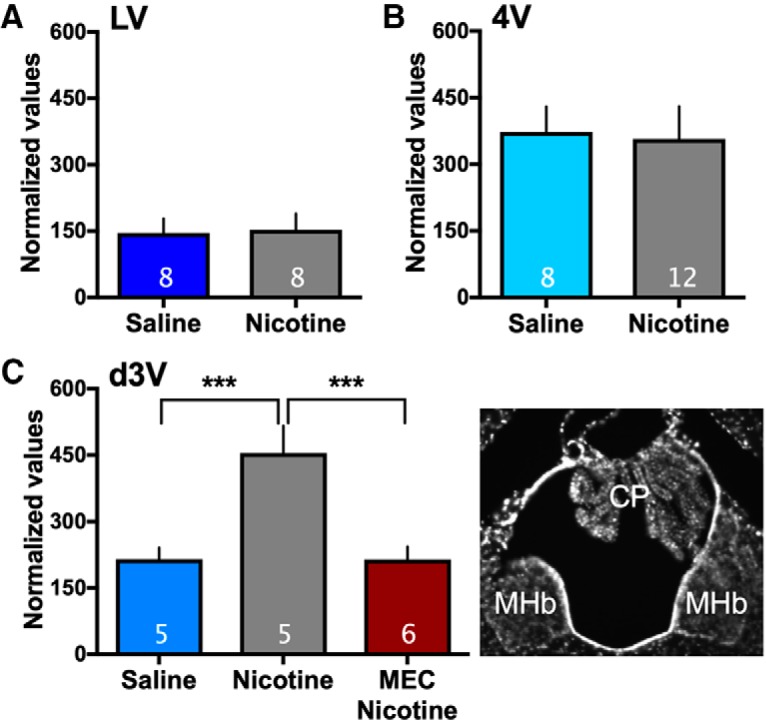
Transthyretin expression in the choroid plexus with saline or nicotine self-administration. Expression of choroid plexus specific transthyretin mRNA was examined in rats self-administering saline or nicotine (*n* = 5–12/group, specific number per group denoted on each bar). ***A***, In the lateral ventricle (LV), differences were not found between groups in transthyretin expression. ***B***, In the fourth ventricle (4V), no differences were found in transthyretin expression. ***C***, In the dorsal third ventricle (d3V), significant upregulation of transthyretin was found with nicotine self-administration, an effect that was reversed with pretreatment of mecamylamine (MEC) before nicotine self-administration; ***p < 0.001. The photomicrograph on the right displays the localization of the choroid plexus (CP) in the d3V, which is in proximity to the habenula (Hb). Central tendency (mean) and variation (SEM) values for each are as follows: *LV - Saline*: 145.9 ± 32.23; *Nicotine*: 152.9 ± 36.57; *4V - Saline*: 372.8 ± 57.27; *Nicotine*: 357.3 ± 73.58; *d3V - Saline*: 214.5 ± 25.81; *Nicotine*: 455.0 ± 61.43; and *Mecamylamine/Nicotine*: 214.3 ± 29.21. For all of the above, expression data were normalized to expression of β-actin. Data represent mean normalized values ± SEM.

To investigate whether nicotine differentially acts on the choroid plexus in a site-specific manner, we examined expression of the choroid plexus-specific protein, transthyretin, in rats self-administering saline or nicotine (for nicotine self-administration data, see Fig. 5). A prior study found that nicotine exposure increases transthyretin mRNA in gross dissections of the hippocampus and surrounding ventricles, as well as the protein in the CSF (Li et al., 2000), but these investigations did not specifically isolate the choroid plexus tissue or examine expression across the choroid plexus ventricular sites. Further, since transthyretin protein is released from the choroid plexus and into the CSF in a dynamic manner, we focused on expression of the mRNA transcript 30-min following the end of the self-administration session with discrete dissections from each of the ventricular locations. Interestingly, a statistically significant increase was found in transthyretin mRNA expression with nicotine self-administration in the d3V, which is localized in proximity to the medial habenula. Further, this increased expression was prevented with pretreatment of mecamylamine (2 mg/kg; one-way ANOVA, *F*_(2,13)_ = 11.31, *p* = 0.0014, *R* ^2^ = 0.6351; Fig. 6*C*). No differences in expression were found in the lateral ventricle (two-sided, unpaired *t* test, *t*_(14)_ = 0.14, *p* = 0.8879, *R* ^2^ = 0.001; Fig. 6*A*) or fourth ventricle (two-sided, unpaired *t* test, *t*_(18)_ = 0.15, *p* = 0.8804, *R* ^2^ = 0.001; Fig. 6*B*) choroid plexus tissues, identifying region-specific effects of nicotine’s actions on this mechanism.

### Differential expression of mir-204 in the d3V choroid plexus with nicotine

**Figure 7.**
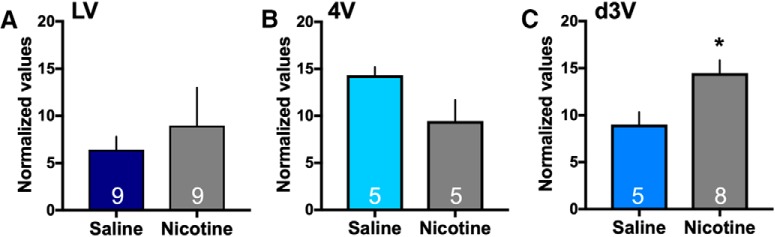
Nicotine mediated changes in expression of mir-204 in the choroid plexus. The expression of mir-204 was examined following saline or nicotine self-administration for each of the choroid plexus sites (*n* = 5–9/group, specific number per group denoted on each bar). ***A***, Differences in expression of mir-204 were not found in the lateral ventricle choroid plexus between the nicotine and saline self-administration groups. Central tendency (mean) and variation (SEM) values for each are as follows: *Saline*: 6.43 ± 1.38 and *Nicotine*: 8.98 ± 4.01. ***B***, In the fourth ventricle choroid plexus, statistically significant group differences were not found. Central tendency and variation: *Saline*: 14.32 ± 0.87 and *Nicotine*: 9.46 ± 2.24. ***C***, In the d3V choroid plexus, nicotine self-administration induced a significant increase in the expression of mir-204 as compared to saline control. Central tendency (mean) and variation (SEM) values for each are as follows: *Saline*: 9.004 ± 1.32 and *Nicotine*: 14.49 ± 1.36; *p < 0.05. Data represent mean normalized values ± SEM.

Given that miRNAs may be involved in the choroid plexus as an alternate mechanism of mediating cellular function, we next examined whether intravenous nicotine self-administration alters miRNA expression in the choroid plexus (for nicotine self-administration data, see Fig. 5). Specifically, mir-204 is localized to the sixth intron of the transient receptor potential melastatin 3 (TRPM3 gene) and is expressed under the control of this gene promotor in high density in the choroid plexus (Oberwinkler et al., 2005; Li et al., 2016). In a preliminary mir-204 gene expression assay (*n* = 3/group), we found that nicotine self-administration induced a significant increase in mir-204 expression in the d3V choroid plexus (two-sided, unpaired *t* test, *t*_(4)_ = 3.98, *p* = 0.0164, *R* ^2^ = 0.798; mean ± SEM: Saline: 10.82 ± 5.19 and Nicotine: 37.53 ± 4.25; *p < 0.05) as compared to saline. However, group differences were not found in the lateral ventricle (two-sided, unpaired *t* test, *t*_(4)_ = 0.797, *p* = 0.4699, *R* ^2^ = 0.137; mean ± SEM: Saline: 36.68 ± 11.80 and Nicotine: 52.97 ± 16.66) or fourth ventricle (two-sided, unpaired *t* test, *t*_(4)_ = 0.185, *p* = 0.8624, *R* ^2^ = 0.009; mean ± SEM: Saline: 53.16 ± 7.31 and Nicotine: 56.62 ± 17.23). While the gene expression assay normalizes to the common housekeeping gene, β-actin, it may also identify other sections of the TRPM3 gene, in addition to mir-204. Therefore, we next used the more selective mir-204 microRNA assay that normalizes to U6, the preferred normalization factor for miRNA analysis in the field, to specifically validate the differences found with the gene assay. In the d3V choroid plexus, a significant increase in mir-204 expression was again evidenced with nicotine self-administration (two-sided, unpaired *t* test, *t*_(11)_ = 2.70, *p* = 0.0103, *R* ^2^ = 0.399; Fig. 7*C*), thus confirming the prior findings. Differences in mir-204 expression were not found between groups in the choroid plexus of the lateral ventricle (two-sided, unpaired *t* test, *t*_(16)_ = 0.60, *p* = 0.555, *R* ^2^ = 0.022) or fourth ventricle (two-sided, unpaired *t* test, *t*_(8)_ = 2.02, *p* = 0.078, *R* ^2^ = 0.339).

### Human choroid plexus expresses nAChRs, transthyretin and mir-204

**Figure 8.**
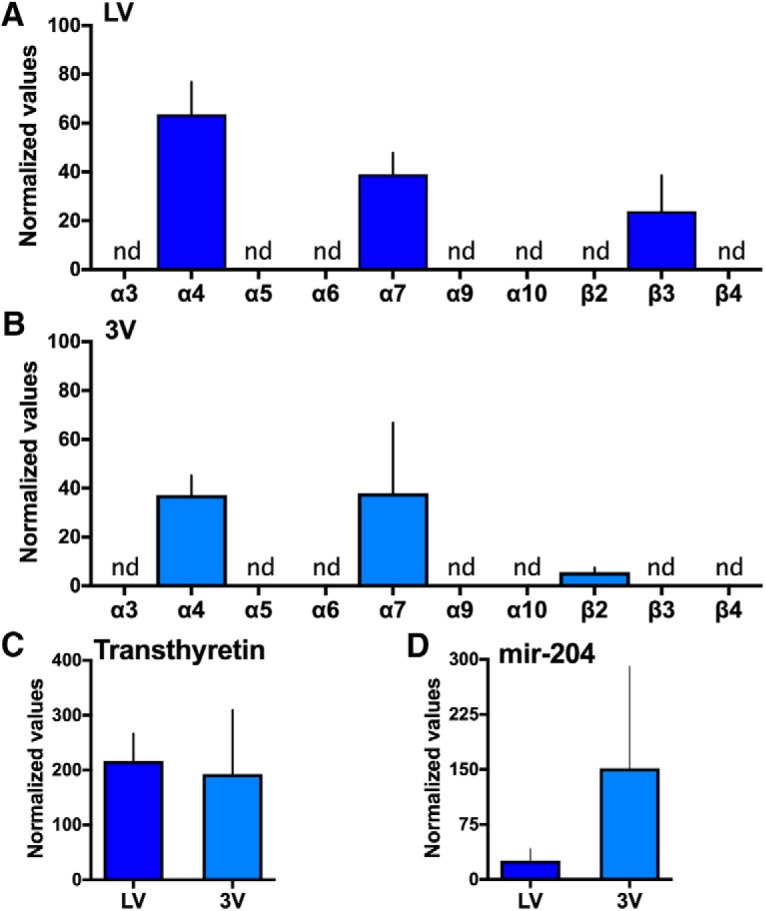
Expression of nAChR subunits, transthyretin and mir-204 in human choroid plexus tissue. Choroid plexus from the lateral and third ventricle (3V) were examined from postmortem humans (*n* = 3 for all analyses shown). ***A***, In the lateral ventricle (LV), mRNA expressions of α4 (*CHRNA4*), α7 (*CHRNA7*), and β3 (*CHRNB3*) were detected. Statistical data are as follows Central tendency (mean) ± variation (SEM), lower 95% confidence interval, upper 95% confidence interval: *α4*: 63.51 ± 13.39, 5.88, 121.1; *α7*: 38.97 ± 8.83, 0.95, 76.98; and *β3*: 23.97 ± 14.55, 0, 86.56. ***B***, In the 3V, mRNA expressions of α4 (*CHRNA4*), α7 (*CHRNA7*), and β2 (*CHRNB2*) were identified. Statistical data are: *α4*: 37.30 ± 7.90, 3.33, 71.28; *α7*: 38.06 ± 28.76, 0, 161.8; and *β2*: 5.62 ± 1.92, 0, 13.86. ***C***, Transthyretin expression was found at similar levels in the lateral and 3V of human tissue. Statistical data (mean ± SEM, lower CI, upper CI) are: *LV*: 216.7 ± 50.10, 1.15, 432.3 and *3V*: 192.6 ± 117.2, 0, 696.8. ***D***, Expression of mir-204 was abundantly localized in the 3V choroid plexus, with lower levels found in the lateral ventricle tissue. Statistical data (mean ± SEM, lower CI, upper CI) are: *LV*: 25.91 ± 16.36, 0, 96.31 and *3V*: 151.5 ± 139.1, 0, 749.9. Expression data were normalized to expression of β-actin (*n* = 3 for each gene/region). For all of the above, the absence of a bar above the denoted nAChR subunit indicates that the mRNA was not detected (nd) based on the predetermined RT-qPCR criteria (Ct value > 35). Data represent mean normalized values ± SEM.

Since nAChR subtype distribution can vary across species (Gotti et al., 1997), we next validated the expression of nAChR subunit mRNA in tissue from humans. Expression of α4, α7, β2 and β3 nAChR subunits were identified, with α4, α7 and β3 localized to the lateral ventricle (Fig. 8*A*) and α4, α7, and β2 localized to the 3V (Fig. 8*B*). Expression of transthyretin (Fig. 8*C*) and mir-204 (Fig. 8*D*) were also found in both the lateral and 3V human choroid plexus tissues.

## Discussion

Together, the current studies reveal that activation of nicotinic receptors during nicotine self-administration induces an upregulation in transthyretin specifically within the d3V choroid plexus. This effect was reversed by the nicotinic receptor antagonist, mecamylamine, demonstrating a direct effect of nicotine on the nAChRs in mediating gene expression. In addition to changes in transthyretin expression, nicotine also site specifically increased the expression of mir-204, a miRNA transcript found in high abundance within the choroid plexus. These effects on the choroid plexus could be attributed to endogenous cholinergic signaling mechanisms within the choroid plexus. Specifically, a subset of epithelial cells was found to express the cholinergic marker ChAT, and nAChR subunits were identified in all of the choroid plexus ventricular sites. Of further note, the specific nAChR subunit expression varied across ventricular regions, providing further evidence of the ability of nicotine to exert site-specific effects on choroid plexus mechanisms. The nAChRs were also shown to be functionally active as nicotine directly led to increased cellular excitability, as evidenced with Ca^2+^ influx. Finally, cholinergic signaling mechanisms were also discovered in human choroid plexus, supporting the translational relevance of the current studies. Taken together, these findings expand our understanding of the extent of cholinergic function in the brain and elucidate a previously unrecognized source of signaling that may be alternately regulated under states of cholinergic dysfunction and/or with pharmacological activation of nAChRs, such as during nicotine dependence.

### Site-specific differences in the choroid plexus

While prior characterization of the choroid plexus has assumed similar function across ventricular sites, recent findings in embryonic mice have suggested differential transcript expression during development (Lun et al., 2015b). The current findings provide novel evidence of region-specific function of the choroid plexus during adulthood, as nicotine self-administration induced differences in gene and miRNA expression selectively in the d3V. The reason for such different actions of nicotine may be attributed to the nAChR subtype expressed within the discrete tissue locations. In the d3V, the α4, α7, β2, and β3 nAChR subtypes were found in the choroid plexus, consistent with expression patterns exhibited in this region in nAChR subunit transgenic mice (GENSAT, 2019). The α4 and β2 subunits can combine together to form a functional receptor subtype, and the β3 subunit has been shown to co-assemble with the α4β2 subtype as well. In contrast, the α7 subunit most often forms a homomeric structure, although some reports suggest the presence of an α7β2 functional subtype (Wu et al., 2016). Moreover, the α4β2 and α7 nAChR subtypes demonstrate functional differences with ligand binding. The α4β2-containing nAChR subtype has a high probability of opening and slower desensitization rate (Li and Steinbach, 2010), whereas the α7 nAChR subtype exhibits higher calcium permeability, lower probability of opening, and rapid desensitization (Williams et al., 2011). Notably, the α4β2 and α7 receptor subtypes have been implicated in learning and memory, drug reinforcement, anxiety, schizophrenia, and immune function (Fowler et al., 2008; Dani, 2015; Wu et al., 2016). The latter function provides an important consideration given that the choroid plexus modulates innate immunity and viral infiltration into the brain (Skok et al., 2005; Vercellino et al., 2008; Ridder et al., 2014; Zhang et al., 2015; Wu et al., 2016). In our Ca^2+^ imaging study, we provide further evidence that functional nAChRs are present in choroid plexus epithelial cells, as nicotine exposure resulted in substantial intracellular Ca^2+^ influx. These studies also documented a subpopulation of cells exhibiting excitability with nicotine application, which parallels the relative proportion of cholinergic epithelial cells found in the choroid plexus in the fluorescence reporter mice. Moreover, the temporal evolution of the Ca^2+^ responses *in vitro* closely mimics the temporal patterns of Ca^2+^ fluxes found with α4β2 nAChRs in different preparations (Vernino et al., 1994; Demuro et al., 2001).

Of further note, we did not detect any significant differences in nAChR subunit mRNA expression with intravenous nicotine self-administration, with the exception of a decrease in β2 subunit expression in the fourth ventricle. In prior studies (Marks et al., 1992; Pauly et al., 1996), nAChR binding has been shown to increase at the cellular membrane during chronic nicotine administration via implanted minipump, whereas limited differences in mRNA expression were evidenced. Since nicotine has been shown to act as a ‘molecular chaperone’ to facilitate the trafficking and insertion of α4-containing and β2-containing nAChR subtypes into the cell membrane (Kuryatov et al., 2005; Srinivasan et al., 2011; Henderson et al., 2014), it is possible that altered expression of nAChRs on the cell membrane could have been induced under the self-administration conditions, an intriguing possibility that will need to be examined in further studies.

### Potential relevance to human disease

The specific effect of nicotine on transthyretin expression within the d3V has several implications. After being released from the choroid plexus into the CSF, transthyretin transports thyroxine and retinol/retinol binding protein throughout the brain. Interestingly, differences in transthyretin levels have been noted in clinical populations. For instance, patients with schizophrenia have been found to have reduced levels of transthyretin and altered nicotinic receptor signaling has been associated with schizophrenia in humans and animal models (Diwan et al., 1998; Wan et al., 2006; Koukouli et al., 2017), suggesting a potential relevance of choroid plexus function in the disease state. With regard to Alzheimer’s disease and dementia, a decrease in transthyretin is correlated with increased risk of severe dementia (Serot et al., 1997). Transthyretin has been proposed to prevent Aβ fibrillogenesis to limit disease progression (Schwarzman et al., 1994; Golabek et al., 1995). Increased nicotine consumption in smokers has also been correlated with decreased risk of Alzheimer’s disease (van Duijn and Hofman, 1991), and in mice, nicotine treatment reduces the presence of insoluble Aβ (Nordberg et al., 2002; Inestrosa et al., 2013). Although prior findings appeared to support the hypothesis that nicotine may act through transthyretin to beneficially modify Alzheimer’s disease pathology (Li et al., 2000), findings from the current study suggest that any beneficial effects of transthyretin would be limited to regions in close proximity to the d3V. Further, it is worthwhile to note that in our analysis of the human tissue, variability was noted in the levels of transcript expression, which could potentially be attributed to individual differences among the subjects (e.g., sex; ethnic background; history of drug use: tobacco, psychostimulants, or alcohol). Moreover, reduced levels of mir-204 have been found in the CSF of patients with frontotemporal dementia (FTD), and as such, this miRNA has been proposed to be a biomarker for the disease state (Nissen et al., 2018). In light of the current findings, such a proposed biomarker approach will need to consider smoking status since nicotine may mitigate the level of mir-204 expression. Further, it will be important in future studies to more directly determine whether mir-204 alters gene expression underlying pathology affecting memory function and whether nicotine could modulate any changes in this regard.

## Conclusions

The current studies identify a previously unrecognized source of cholinergic signaling within the brain and identify the direct mechanism through which nicotine acts on this tissue to alter function. Findings derived from the human choroid plexus further support the translational relevance of the rodent studies. In addition to important implications for tobacco/nicotine dependence, the current findings elucidate a mechanism through which cholinergic signaling may impact global brain function via release of factors into the CSF, which could further our understanding of multiple disease states characterized by cholinergic dysfunction, and these findings may also have important implications for patients prescribed pharmacotherapeutics with actions on the cholinergic system.

## Synthesis

Reviewing Editor: Yavin Shaham, NIDA-IRP/NIH Decisions are customarily a result of the Reviewing Editor and the peer reviewers coming together and discussing their recommendations until a consensus is reached. When revisions are invited, a fact-based synthesis statement explaining their decision and outlining what is needed to prepare a revision will be listed below. The following reviewer(s) agreed to reveal their identity: Cassandra Gipson, Jill Turner.

The reviewers found significant merit in the study but have made several comments that should be addressed in the revision. Based on their comment, we do not think that additional experimental data are necessary for a successful revision. I enclose below the individual comments of the reviewers and in addition to their comments, also address the comments below:

1. Add 2-3 informative sub-headings to the Discussion section to orient the readers on the topics being discussed

2. Change ‘volitional nicotine intake’ to ‘nicotine self-administration’ in different places in the text

3. Include the behavioral data for nicotine self-administration in the figures

REVIEWER 1

The manuscript titled “Nicotine Acts on Cholinergic Signaling Mechanisms to Directly Modulate Choroid Plexus Function” (eN-NWR-0051-19) is a novel contribution to our understanding of the mechanisms by which nicotine impacts choroid plexus function. This manuscript utilizes tissue from both rodent and human choroid plexus, adding translational value. An array of techniques is used, including Ca2+ imaging to identify functional activation of epithelial cells within the choroid plexus by nicotine, as well as use of transgenic mice to identify cholinergic signaling mechanisms within the choroid plexus, among others. Overall, the manuscript is well written, utilizes a novel array of techniques to characterize mechanisms by which nicotine acts in the choroid plexus across species in a region-specific manner, and contributes significantly to the field. Minor points are listed below:

1. The authors state that “choroid plexus factors” regulate neurogenesis among other things, however, this statement is vague. More specificity regarding what these factors are would be helpful.

2. Justification for examining specifically transthyretin and mir-204 within choroid plexus is not provided in the introduction (why these targets and not others?).

3. The total number of rats and mice should be included in the Animals subsection of the methods section

4. Acronyms should be properly defined in the abstract.

5. Given that some tissue was pooled in some experiments (e.g., cell culture), number of subjects per group in the bars of the figures would be helpful.

6. Error bars are large in Figure 7, from the human tissue experiments (e.g., for miR-204 in the 3rd ventricle). Is this due to lack of power?

7. N=3 per group in nicotine/saline self-administration experiment seems unlikely to achieve appropriate power. As well, self-administration data would be helpful in Figure 6.

8. Given that rats were exposed to different doses of nicotine for a different number of sessions at each dose, a timeline would be helpful to follow the behavioral protocol utilized. As well, 0.12 mg kg-1 is a very high dose of intravenous nicotine, and justification for this dose is needed in the methods section.

REVIEWER 2

This is a generally well-written manuscript outlining some exciting findings regarding nicotine exposures effect in vivo and in vitro on cells of the choroid plexus. The authors include a variety of in vivo and in vitro techniques to convincingly demonstrate that choroid plexus cells express functional nicotinic receptors and that there are distinct subtypes expressed depending up on the specific ventricle examined. The authors then go on to re-examine their findings in human brain choroid plexus tissues, lending translational relevance to their line of inquiry. While these are exciting data, given that little is known regarding nicotinic signaling in this structure, there are a few fairly minor criticisms regarding some of the experiments.

Experimental Comments:

1) In the materials and methods on page 10, please expand more details on the how choroid plexus was dissected (freehand? Micropunch?). This is shown somewhat later in the figures, but please also describe more extensively here.

2) Regarding the RNA Analysis of the mi-204, please go into more detail either in the methods or the results regarding why two methods were used. While this reviewer assumes this is due to normalization to a co-expressed miRNA to serve as a house-keeping gene, it is not explicitly addressed.

3) In the TIRF results, the authors used a concentration of 30uM nicotine in the bath. This is quite a high concentration, with most physiological experiments using doses ranging from 300nM to 1uM at the high end. Is there any rationale for this dose, or why lower doses were not utilized?

4) Additionally, do the authors' know whether the Ca2+ flux following nicotinic stimulation is due to direct passage through the nAChRs or whether there is a Ca2+ induced internal Ca2+ release component at play? Does the sub-cellular localization of these signals suggest one or the other?

5) For the RNA analyses of dissected choroid plexus, it would be good to have controls demonstrating that the nicotinic receptor expression is different from the surrounding tissues. Eg, for the lateral ventricles, perhaps take part of the striatum or hippocampus and compare?

6) Two seminal papers (Marks et al, JNeurosci 1992 and Pauly et al, JPET 1996) both demonstrated that chronic nicotine increased nAChR binding, but had no impact on mRNA levels for the nAChR subunits. This makes the findings outlined in Figure 4 consistent with the literature. However, this now begs the question of whether there are protein changes or increased assembly of the nAChRs following SA nicotine, as indicated via radioligand binding. With only the mRNA data, it is difficult to say.

7) The mRNA expression data for the miRNA is currently and N of 3. In the lateral ventricle, there looks to be an upward trend following nicotine. Increased power for these analyses may indicated that more than the d3V expresses mir204.

Prose Comments:

1) On page 4 of the introduction, the last sentence of the top paragraph is missing a ‘be’ prior to ‘localized’

2) On page 5 in the materials and methods, the last sentence needs to include the “Author University”

## Author Response

Dear Editor and Reviewers,

We would like to thank you for your critical analysis and thoughtful comments of the manuscript. We greatly appreciate the positive reviews indicating the ‘significant merit in the study’ and further notation that this manuscript ‘contributes significantly to the field’. In response to the main concern regarding mir-204 analysis with n=3 for the lateral and fourth ventricles, we have conducted an additional experiment to analyze the choroid plexus tissue with the more specific probe for mir-204, and the findings are consistent with our prior conclusions. These new data have been included in the manuscript, in addition to the following in response to each editor and reviewer comment. Significant changes in the manuscript have been denoted with blue text.

Editor Comments:

1. Add 2-3 informative sub-headings to the Discussion section to orient the readers on the topics being discussed

Response: Sub-headings have now been included.

2. Change ‘volitional nicotine intake’ to ‘nicotine self-administration’ in different places in the text

Response: This change has been incorporated accordingly.

3. Include the behavioral data for nicotine self-administration in the figures

Response: The behavioral data for nicotine self-administration have been added. Since some tissue was analyzed across studies, all of the behavioral data have been included in a separate figure to avoid duplication of data across figures.

REVIEWER 1

1. The authors state that “choroid plexus factors” regulate neurogenesis among other things, however, this statement is vague. More specificity regarding what these factors are would be helpful.

Response: We have provided further specific information for the choroid plexus factors implicated in mediating neurogenesis and Alzheimer's disease.

2. Justification for examining specifically transthyretin and mir-204 within choroid plexus is not provided in the introduction (why these targets and not others?).

Response: We apologize for this oversight. This information has now been incorporated into the introduction.

3. The total number of rats and mice should be included in the Animals subsection of the methods section

Response: This information has been added to the Animals subsection of the methods.

4. Acronyms should be properly defined in the abstract.

Response: This information has been corrected in the abstract.

5. Given that some tissue was pooled in some experiments (e.g., cell culture), number of subjects per group in the bars of the figures would be helpful.

Response: The number of subjects per group has been more clearly identified on the bars of the graphs as requested.

6. Error bars are large in Figure 7, from the human tissue experiments (e.g., for miR-204 in the 3rd ventricle). Is this due to lack of power?

Response: We analyzed samples from 3 humans. The reasons for such individual differences likely lies in the differences among the humans subjects, which may be explained by sex (2 males, 1 female), ethnic background, drug use status (subjects varied with no reported drugs, tobacco use, psychostimulant (cocaine, methamphetamine) co-use with tobacco, alcoholism, or other unknown factors that may mediate expression (e.g., obesity, deficient nutritional status with homelessness). The goal of this analysis was to examine whether humans expressed nAChR subunits, transthyretin and mir-204, or not, and thus, statistical analysis was not performed (which is why power was not a factor). Information has been added to the discussion to address this point.

7. N=3 per group in nicotine/saline self-administration experiment seems unlikely to achieve appropriate power. As well, self-administration data would be helpful in Figure 6.

Response: We have replicated this study for the lateral and fourth ventricles with the more specific miRNA assay and the ns per group are now 5-9. As in the gene expression assay, no differences were found between groups for the lateral and fourth ventricle locations. The self-administration data has been added for the nicotine groups.

8. Given that rats were exposed to different doses of nicotine for a different number of sessions at each dose, a timeline would be helpful to follow the behavioral protocol utilized. As well, 0.12 mg kg-1 is a very high dose of intravenous nicotine, and justification for this dose is needed in the methods section.

Response: This sequence of testing has been further clarified within the methods. With regard to the rational, we designed the experiment with the transition to this moderately-high dose to have the rats actively titrate their behavior to obtain the desired amount of nicotine. In a prior study (Fowler et al., 2011), we found that rats will titrate their nicotine intake to a similar level across the dose range of 0.06 - 0.18 mg kg-1 per infusion, and given the localization of the dorsal third ventricle choroid plexus in juxtaposition to the medial habenula, which is preferentially activated at higher nicotine doses (Fowler et al., 2011; Frahm et al., 2011), we hypothesized that this dose may be particularly relevant to reveal functionally significant processes potentially mitigating drug intake.

REVIEWER 2

1) In the materials and methods on page 10, please expand more details on the how choroid plexus was dissected (freehand? Micropunch?). This is shown somewhat later in the figures, but please also describe more extensively here.

Response: We apologize for the lack of clarity on the dissection method. Details regarding the freehand dissections for fresh tissue have now been extensively described in the methods.

2) Regarding the RNA Analysis of the mi-204, please go into more detail either in the methods or the results regarding why two methods were used. While this reviewer assumes this is due to normalization to a co-expressed miRNA to serve as a house-keeping gene, it is not explicitly addressed.

Response: In our initial screen, we used the mir-204 gene expression assay, which contains mir-204 in addition to other regions of the TRPM3 gene; this allowed us to use β-actin as a housekeeping control, consistent with the other genes analyzed. To verify the expression differences found in the dorsal third ventricle, we then used the mir-204 assay with U6 as the housekeeping control based on the premise that U6 is more acceptable in the field for miRNA normalization. However, this point is still debatable, as we don't know if U6 expression changes with nicotine self-administration. Thus, we have retained both analyses to provide experimental rigor for the findings. We recognize that this may be confusing for the reader with both graphs presented, so we have now included the results for the gene expression assay in the text, and further, we have added additional analyses for the mir-204 specific assay (with the U6 normalization) in lateral and fourth ventricle choroid plexus tissue, which are now represented in the graphs for Figure 6.

3) In the TIRF results, the authors used a concentration of 30uM nicotine in the bath. This is quite a high concentration, with most physiological experiments using doses ranging from 300nM to 1uM at the high end. Is there any rationale for this dose, or why lower doses were not utilized?

Response: These set of experiments were performed to substantiate the presence of functional nAChRs in the plasma membrane of cultured cells by monitoring agonist induced Ca2+-dependent fluorescence signals. The EC50 for nicotine/acetylcholine for the different neuronal nAChRs subtypes, range from ~2 to 30 μM (Demuro et al., 2001; Demuro and Parker, 2005). As our rational was to generate a robust response by activating all the possible nAChRs subtypes present in the cells we performed experiments using 30 μM nicotine.

4) Additionally, do the authors' know whether the Ca2+ flux following nicotinic stimulation is due to direct passage through the nAChRs or whether there is a Ca2+ induced internal Ca2+ release component at play? Does the sub-cellular localization of these signals suggest one or the other?

Response: The partial permeability to Ca2+ for nAChRs subtypes, range from 0.8 to 4 pS. This relatively small conductance allows a large flux of Ca2+ into the cells which, by binding to the Ca2+ sensitive dye is amplified to become a robust fluorescence signal. As such, in the TIRF condition, it is now possible to monitor Ca2+ flux trough the opening of a single nAChRs (Demuro and Parker, 2005; Demuro and Parker, 2006). Although we cannot exclude the possibility of other components contributing to the cytosolic Ca2+ rise, such as CICR through RyRs or calcium influx through store-operated Ca2+ channels, the temporal evolution and the time to response obtained in these experiments strongly suggest that the fluorescence signal is due to the opening nAChRs in the plasma membrane of these cells.

5) For the RNA analyses of dissected choroid plexus, it would be good to have controls demonstrating that the nicotinic receptor expression is different from the surrounding tissues. Eg, for the lateral ventricles, perhaps take part of the striatum or hippocampus and compare?

Response: With our fresh tissue dissection technique, we validate at the time of dissection the lack of potentially contaminating tissues. The choroid plexus exhibits a distinct morphological structure, allowing for it to be easily dissected by free hand and discerned from surrounding tissue under the microscope. In response to this concern, we have now provided more extensive details for our dissection technique, in which it is noted that we validate the tissue obtained with dissection as only having the morphological structure of the choroid plexus under the microscope. Examining the presence of RNA in other tissues would not provide the evidence requested, as similar nAChR expression would be expected in these locations based on the evidence in the literature. To provide further confidence in our findings, we also now note the corroborating expression patterns found in transgenic nAChR subunit cre-driver mice in the GENSAT database.

6) Two seminal papers (Marks et al, JNeurosci 1992 and Pauly et al, JPET 1996) both demonstrated that chronic nicotine increased nAChR binding, but had no impact on mRNA levels for the nAChR subunits. This makes the findings outlined in Figure 4 consistent with the literature. However, this now begs the question of whether there are protein changes or increased assembly of the nAChRs following SA nicotine, as indicated via radioligand binding. With only the mRNA data, it is difficult to say.

Response: Yes, we agree that this is an important point, although we did find a significant decrease in β2 mRNA in the fourth ventricle choroid plexus with nicotine self-administration. However, since no consistently reliable, commercially available antibodies are present for the specific nAChR subunits, we are not able to confidently assess levels of protein expression for each nAChR subunit. This consideration for the interpretation of the findings has been incorporated into the discussion.

7) The mRNA expression data for the miRNA is currently and N of 3. In the lateral ventricle, there looks to be an upward trend following nicotine. Increased power for these analyses may indicated that more than the d3V expresses mir204.

Response: We have replicated this study for the lateral and fourth ventricles with the more specific miRNA assay. The Ns per group are 5-9, with the lateral ventricle analysis containing 9 per group to more fully address the concern of power based on the reviewer comments. As in the gene expression assay, no differences were found between groups.

Prose Comments:

1) On page 4 of the introduction, the last sentence of the top paragraph is missing a “be” prior to “localized”

Response: The sentence has been corrected.

2) On page 5 in the materials and methods, the last sentence needs to include the “Author University”

Response: Thank you for your thorough reading of the manuscript. This formatting was a submission requirement for eNeuro and will be modified accordingly following manuscript acceptance.
